# Metabolite Profiling of Wheat Seedlings Induced by Chitosan: Revelation of the Enhanced Carbon and Nitrogen Metabolism

**DOI:** 10.3389/fpls.2017.02017

**Published:** 2017-11-28

**Authors:** Xiaoqian Zhang, Kecheng Li, Ronge Xing, Song Liu, Pengcheng Li

**Affiliations:** ^1^Key Laboratory of Experimental Marine Biology, Institute of Oceanology, Chinese Academy of Sciences, Qingdao, China; ^2^Laboratory for Marine Drugs and Bioproducts, Qingdao National Laboratory for Marine Science and Technology, Qingdao, China; ^3^University of Chinese Academy of Sciences, Beijing, China

**Keywords:** chitosan, metabolic profiling, carbon metabolism, nitrogen assimilation, wheat seedlings

## Abstract

Chitosan plays an important role in regulating growth and eliciting defense in many plant species. However, the exact metabolic response of plants to chitosan is still not clear. The present study performed an integrative analysis of metabolite profiles in chitosan-treated wheat seedlings and further investigated the response of enzyme activities and transcript expression related to the primary carbon (C) and nitrogen (N) metabolism. Metabolite profiling revealed that chitosan could induce significant difference of organic acids, sugars and amino acids in leaves of wheat seedlings. A higher accumulation of sucrose content was observed after chitosan treatment, accompanied by an increase in sucrose phosphate synthase (SPS) and fructose 1, 6-2 phosphatase (FBPase) activities as well as an up-regulation of relative expression level. Several metabolites associated with tricarboxylic acid (TCA) cycle, including oxaloacetate and malate, were also improved along with an elevation of phosphoenolpyruvate carboxylase (PEPC) and pyruvate dehydrogenase (PDH) activities. On the other hand, chitosan could also enhance the N reduction and N assimilation. Glutamate, aspartate and some other amino acids were higher in chitosan-treated plants, accompanied by the activation of key enzymes of N reduction and glutamine synthetase/glutamate synthase (GS/GOGAT) cycle. Together, these results suggested a pleiotropic modulation of carbon and nitrogen metabolism in wheat seedlings induced by chitosan and provided a significant insight into the metabolic mechanism of plants in response to chitosan for the first time, and it would give a basic guidance for the future application of chitosan in agriculture.

## Introduction

Conventional crop production is increasingly being challenged by various problems such as decreased soil fertility and pollution due to the use of hazardous chemical pesticides and fertilizers at a global scale (Vassilev et al., 2015). In the same time, there has been mass awareness of quality and safety of food production. This situation escalates public concerns regarding the use of eco-friendly growth-regulators which contribute to not only improve plant growth and development but also produce organic greens (Brown and Saa, 2015). Recently, biological polysaccharides have attracted increasing interest as a natural plant growth regulator. Many recent findings clearly suggested that exogenous application of biological polysaccharides in a variety of crops played a positive role in growth, development and defense against biotic and abiotic stress, which had huge potential in the future sustainable crop production (Rahman et al., 2013).

Chitosan is a natural linear polysaccharide, derived from chitin that is often considered as the second most abundant polysaccharide in nature following plant cellulose and mainly occurs as a structural component in the cell walls of fungi and yeasts and in the exoskeletons of insects, nematodes and arthropods (e.g., crabs, crawfish, lobsters, and shrimps). As for the chemical structure of chitosan, it is mainly made up of D-glucosamine (GlcN) and partially of *N*-acetyl-D-glucosamine (GlcNAc), linked by β-1,4 glycosidic bonds. In combination with its non-toxicity, biocompatibility and biodegradability, chitosan exhibits numerous interesting physicochemical and biological properties, which make it suitable for use in many fields (Khor and Lim, 2003; Muzzarelli, 2010). In particular, those chitosan with low molecular weight shows much improved water solubility and better bioactivities (Kim and Rajapakse, 2005; Aam et al., 2010; Xia et al., 2011). In agriculture, chitosan have been used to activate plant innate immunity against plant diseases (Khairullin et al., 2001; Munoz et al., 2009; Yin et al., 2010). Yin et al. (2013) reported that chitosan elicited plant defense against *Sclerotinia sclerotiorum* via the jasmonic acid–ethylene (JA/ET) signal pathway, and NO and H_2_O_2_ participated in this signaling pathway in *Brassica napus.* Apart from biotic stress, it was also reported that chitosan could improve the abiotic stress tolerance of plants. Spraying chitooligosaccharide onto plant leaves was shown to promote the cadmium and salt stress tolerance under greenhouse conditions (Zou et al., 2015; Zong et al., 2017). On the other hand, chitosan has attracted wide interest as a potential bio-stimulator (Malerba and Cerana, 2016). It has been reported that chitosan could promote the growth of orchid tissue (Nge et al., 2006), improve the photosynthesis rate and stomatal conductance in the leaves of maize (Khan et al., 2002), enhance the plant parameters and fruit yield of okra (Mondal et al., 2012), stimulate the seed germination and protocorm development (Kananont et al., 2010), increase the field yield of wheat (Wang et al., 2015), and induce the synthesis of indole-3-acetic acid (IAA) in tobacco (Guo et al., 2009). Recently, the transcriptional response to a chitin oligosaccharide in *Arabidopsis* was conducted by Winkler et al. (2017), suggesting that chitin could also induce the expression of genes related to vegetative growth, development and primary metabolism. Consequently, based on the obviously promoting effect on plants, a variety of agricultural applications of chitosan have been developed recently. Taking China as an example, currently, there are more than 50 chitosan-based bio-products with official issued certificates in agriculture. However, as for the bioactivity of chitosan on promoting plant growth, previous studies mostly focused on the apparent effects of chitosan on the plant physiology and growth characteristics. There are few reports about the metabolic response mechanism of plant to chitosan. It has attracted wide interests in understanding the mechanisms of chitosan-induced growth-promoting effect, which will provide a considerable agronomic benefit for future large-scale application of chitosan in crop production.

The biomass accumulation in plant growth actually can be regarded as the ultimate performance of its metabolic pathways (Meyer et al., 2007). Carbon (C) and nitrogen (N) are both primary nutrients for plant growth and crop yields (Coruzzi and Bush, 2001; Miyagawa et al., 2001). It is believed that C and N metabolism are closely connected with each other in almost every metabolic pathway of plants. The coordination and integration of C and N metabolism, such as Calvin cycle, sucrose metabolism, glycolysis, TCA cycle and the central N metabolism, are vital for the improvement of plant growth and development. C metabolism could supply the reducing power, ATP and C skeletons for N assimilation. In turn, photosynthetic carbon fixation requires nitrogen to synthesize proteins that enhance electron transport and catalyze photosynthetic reactions (Nunes-Nesi et al., 2010). Actually, metabolites play a crucial role in regulating those biochemical processes in plant growth (Vincentz et al., 1993), such as the nitrate induction of NR and enzymes involved in organic acid synthesis (Foyer et al., 2003). Thus, the identification and analysis of metabolites would contribute to a comprehensive insight into metabolic mechanisms of plants under internal or external stimulating conditions. However, the metabolism pathway in plants is an interconnected network consisted of mostly enzyme catalyzed reactions that occur in a cell. So many reactions have a common substrate or product, and many metabolites also influence the activities of enzymes that are directly or indirectly involved in their metabolism (Sweetlove, 2008). In addition, it had been reported that the responses of plants to developmental and environmental changes were not synchronous at metabolites, transcripts and protein levels. The transcriptional response was faster than the changes in metabolite profiles and enzyme activities (Gibon et al., 2004, 2006). Hence, an integrative analysis of metabolite profiling, transcripts and enzyme activities is necessary to understand the comprehensive response mechanisms of plants, which will provide much more actual information about the metabolic pathways involved.

Wheat is one of the major food crops in the world. To investigate the chitosan-triggered metabolic responses mechanism in wheat seedlings, three chitosan oligosaccharides, including (GlcN)_6_, (GlcN)_7_, and (GlcN)_8_, were applied which were reported more effective in promoting plant growth by Zhang et al. (2016). We first analyzed the effects of three chitosan fragments on primary metabolic pathways of wheat seedlings using metabolite profiling. Then the metabolic responses of wheat seedlings to chitosan, including photosynthetic carbon metabolism, glycolysis, TCA cycle and central N metabolism, were further investigated at transcripts and enzyme activities levels. This article provided a comprehensive study on the metabolic regulation mechanism of chitosan on plants for the first time, and it would give a basic guidance for the future application of chitosan in agriculture.

## Materials and Methods

### Materials

Winter wheat (*Triticum aestivum* L.) Jimai 22, released by Crop Research Institute, Shandong Academy of Agricultural Sciences in 2006, was used in the present study and applied by China national seed group CO., LTD. (Kong et al., 2010). Chitohexaose (GlcN)_6_ (≥98%), chitoheptaose (GlcN)_7_ (≥93%) and chitooctaose (GlcN)_8_ (≥90%) were prepared according to Li et al. (2012, 2013).

### Plant Growth and Treatments

The present study was conducted with wheat seeds. The growth conditions of wheat seedlings were set according to Zhang et al. (2016). After being sterilized, 30 wheat seeds were transferred to a Petri dish with moist gauze for germination at 25°C for 24 h in the dark. Then, germinated seeds were individually transferred to 30 Petri dishes with nylon mesh and each Petri dish contained 30 seeds. Wheat seedlings were applied in Hoagland solution in a growth incubator with a light intensity of 800 mol m^-2^ s^-1^, a day/night cycle of 14 h/10 h at 25°C/15°C, respectively, and the relative humidity was controlled at 70% (Zou et al., 2015). Hoagland solution in Petri dish was replaced every other day up to two leaves stage. When the second leaves of wheat seedlings were fully expanded, the wheat seedlings were separated as four independent treatment groups and each treatment group contained six biological replicates. Chitosan-treated groups were sprayed with 15 mg/L (GlcN)_6_, (GlcN)_7_, and (GlcN)_8_, respectively, and the control plants (CK) were treated with distilled water. After 7 days treatment, 25 second functional leaves of wheat seedlings from each 6 replications in the control and chitosan-treated groups were selected randomly in the morning, which were frozen rapidly with liquid N_2_ and stored at 80°C for the determination of the metabolites, enzyme activities and the expression of relative genes.

### Determination of Metabolites

Frozen leaves of wheat seedlings from each biological replicate were homogenized using a mortar and pestle with liquid nitrogen. The metabolites of the leaves (60 ± 1 mg of fresh weight) were extracted with 0.48 mL of 75% methanol, then 24 μL of adonitol was added to the sample as internal standard. Secondly, the samples were homogenized and centrifuged for 15 min at 13000 rpm. The supernatants from each sample were taken and dried in a vacuum concentrator without heating and then dissolved with 60 μL of methoxy amination hydrochloride (20 mg/mL in pyridine) to be incubated for 30 min at 80°C. Then, the sample aliquots were further incubated with 80 μL of the *N*-methyl-*N*-(trimethylsilyl)trifluoroacetamide (BSTFA) for 2 h at 70°C. Finally, the mixture was used for GC-TOF-MS analysis (Dunn et al., 2011). The separation and quantification of organic acids, carbohydrates, and amino acids were performed by Agilent 7890 gas chromatograph system coupled with a Pegasus HT time-of-flight mass spectrometer according to Wu et al. (2013). Nitrate was quantified by the method described by Dutilleul et al. (2005). The contents of ammonium were measured by the phenol hypochlorite assay (Tercé-Laforgue et al., 2004).

### Enzyme Assays

The frozen leaves were ground to powder with liquid N_2_ and then used to determine the enzyme activities. For the measurement of Rubisco activity, 0.1 g plant tissues were homogenized in an extraction buffer containing 100 mM HEPES-KOH (pH 8.0). The total activities were measured with a spectrophotometer at 340 nm, and one unit of enzyme activity was defined as 1 nmol of NADH oxidation per min at 25°C (Sharkey et al., 1991). FBPase activity was assayed according to the method reported by Hurry et al. (1995). The reaction was initiated by the addition of 0.1 mM Fru-1,6bP. SPS and SS activities were measured according to the method of Heim et al. (1993) and Klann et al. (1993). The measurement of the PEPC activity was conducted with an adapted method reported by Shi et al. (2015). 0.1 gram of leaves was homogenized with 1 mL of 200 mM ice-cold HEPES-NaOH buffer (pH 7.0). The homogenates were then centrifuged at 4°C for 10 min at 8000 *g*, and the supernatants were used for the determination of PEPC activity. HK, PK, IDH, and GOT activities were determined with the methods adapted from Gibon et al. (2004). For MDH, its activity was assayed according to the method reported by Jenner et al. (2001). Briefly, the extracts were added into 1mL of reaction buffer (pH 7.2) containing 50 mM TES-NaOH, 5 mM MgCl_2_, 0.2 mM NAD, and 0.05% Triton X-100. The reaction was started by the addition of 1 mM OAA and the absorbance was read at 340 nm. The NR activity was measured as described by Ferrario-Méry et al. (1998) and NR activity was expressed as μmol h^-1^ g^-1^ FW. GS, GDH and GOGAT activities was measured following the method described by Xu et al. (2014). PDH was extracted with 100 mM Tris-HCl buffer (PH 6.5) containing 1% PVP and 2 mM DTT, and the measurement of PDH was conducted exactly as described by Mustroph and Albrecht (2003).

### Real-Time Quantitative PCR (RT-qPCR) Expression Analysis

Total RNA was extracted from the leaves of wheat seedlings in the chitosan groups and control group with RNAprep Pure Plant Kit (Qiagen) according to the manufacturer’s instructions. The synthesis of the first-strand cDNA and the quantitative real-time RT-PCR were operated as described by our prior report (Zou et al., 2015). For the RT-qPCR analysis, four biological replicates and three technical replicates per sample were made. The expression levels of genes were analyzed using the comparative threshold cycle method (2^-ΔΔC_t_^) and β*-actin* was used as a reference gene in each experiment (Pfaffl, 2001; Bernardo et al., 2007). In addition, all relative gene-specific primers were showed in Supplementary Table S1.

### Data Analysis

As for the metabolomics data analysis, the Chroma TOF4.3X software (LECO) and LECO-Fiehn Rtx5 database were used for peak identification and integration of the peak area. The SIMCA software package (V14.1, MKS Data Analytics Solutions, Umea, Sweden) was used for multivariate analysis, including PCA and orthogonal partial least squares discriminant analysis (OPLS-DA). The identification of differentially expressed metabolites was performed by the VIP values (VIP > 1) of OPLS-DA combined with Student’s *t*-test (*t*-test) (*P* ≤ 0.05). Statistical analyses of the data of the metabolites, enzymes activities and transcript levels were performed using ANOVA analysis and Duncan’s multiple range tests (*P* < 0.05) by SPSS (version 19.0).

## Results

### Metabolite Profiling of the Chitosan-Treated Wheat Seedlings

**FIGURE 1:**
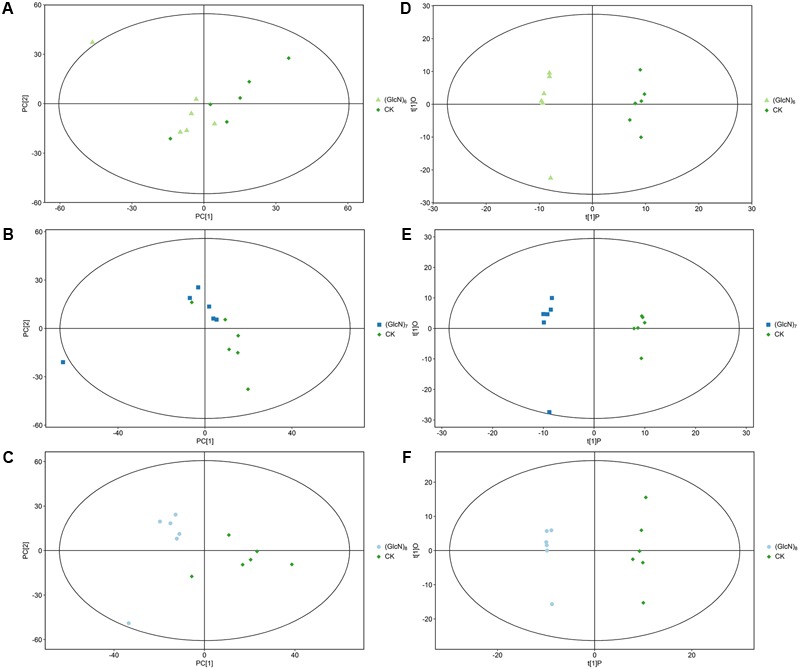
Principal component analysis score map **(A–C)** and OPLS-DA score plots **(D–F)** of the metabolite profiles of different chitosan fragments treatment groups (six biological replicates). **(A,D)** CK-(GlcN)_6_; **(B,E)** CK-(GlcN)_7_; **(C,F)** CK-(GlcN)_8_.

Three most effective chitosan fragments in promoting plant growth, (GlcN)_6_, (GlcN)_7_, and (GlcN)_8_ were used in the present experiments. The growth parameters of 21-day-old wheat seedlings treated with (GlcN)_6_, (GlcN)_7_ and (GlcN)_8_ were measured and showed significant variation (Supplementary Table S2). In order to explore the chitosan-triggered metabolic responses mechanism in wheat seedlings, the metabolite profiling experiments were accordingly carried out, and the multivariate statistical tools were applied to analyze the metabolite datasets. The PCA of the metabolite profiles of different experimental groups is shown in **Figures 1A–C**. The R^2^X values, which represent the explanatory variable in the PCA model, were 0.516, 0.541, and 0.544 in (GlcN)_6_, (GlcN)_7_, and (GlcN)_8_ treatment groups, respectively. **Figures 1D–F** further displayed the OPLS-DA results. The score scatter plots of three experimental groups were all inside the 95% Hotelling T^2^ ellipse. Obvious separations were found in groups of CK-(GlcN)_6_, CK-(GlcN)_7_, and CK-(GlcN)_8_, indicating that the OPLS-DA model can be used to identify the difference between the CK and chitosan-treated groups. The validity of the OPLS-DA model for three treatment groups was further verified using the permutation tests (Supplementary Figures S1A–C). The Q^2^Y values in three experimental groups of CK-(GlcN)_6_, CK-(GlcN)_7_, and CK-(GlcN)_8_ were 0.97, 0.96, and 0.94, respectively (Supplementary Figures S1A–C), which indicated that the model was stable and reliable.

**FIGURE 2:**
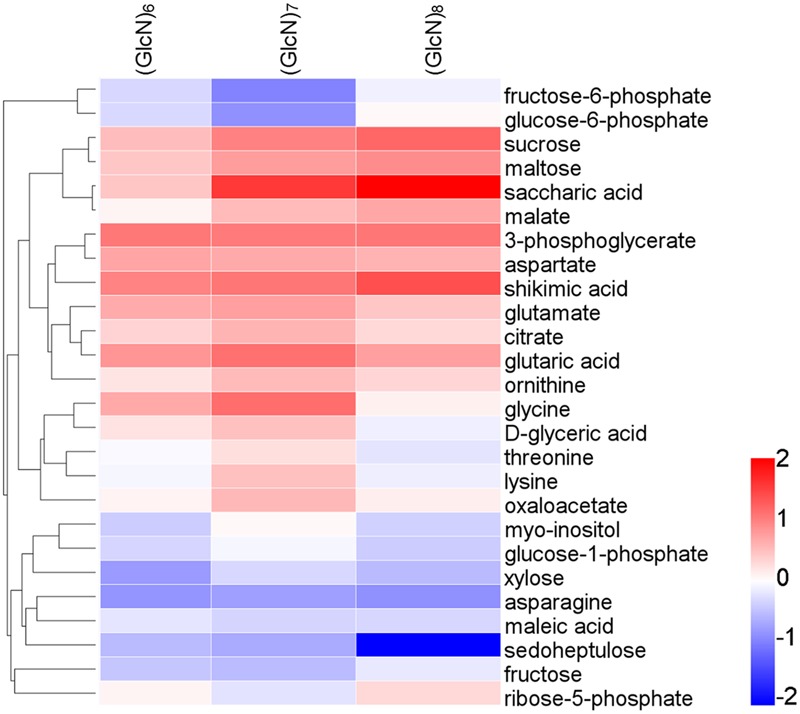
Changes of metabolite levels after spraying wheat seedlings with (GlcN)_6_, (GlcN)_7_, and (GlcN)_8_. Metabolite levels are normalized on the average level in CK, transformed to log_2_ ratios, and then converted to a false color scale as indicated in the legend scale (six biological replicates). This procedure means that metabolites that increase in chitosan-treated seedlings are colored in red, and metabolites decreased in chitosan-treated seedlings are colored in blue. Metabolites with no change are colored in gray.

Furthermore, in (GlcN)_6_, (GlcN)_7_, and (GlcN)_8_ treatment groups, 29, 55, and 48 significantly changed metabolites [variable importance in the projection (VIP) > 1 and *P* < 0.05] were identified, respectively (Supplementary Tables S3–S5). It seemed that the majority of significantly different metabolites were involved in primary C and N metabolism. The levels of xylose, D-altrose, erythrose, 3-PGA, aspartate, asparagine and valine were significantly affected by (GlcN)_6_ in wheat seedlings. Moreover, some significantly changed metabolites related to primary C and N metabolism responded similarly to (GlcN)_7_ and (GlcN)_8_, such as malate, oxaloacetate, 3-PGA, sucrose, maltose, and aspartate. Apart from them, (GlcN)_7_ also affected the accumulation of fructose 6-phosphate (Fru-6P), glucose 6-phosphate (Glc-6P), trehalose 6-phosphate, sedoheptulose, lysine, threonine, asparagine, and glutamate in leaves of wheat seedlings. However, fructose, fucose, erythrose, aconitate, glucose-1-phosphate and ribose-5-phosphate contents were significantly increased in (GlcN)_8_-treated group. In order to make a more sensitive visualization of the metabolites changes in three chitosan fragments treatments, a hot map was generated to display the exactly metabolites changes involved in primary C and N metabolism, including key organic acids, major carbohydrates and amino acids (**Figure 2**). In addition, based on the significantly changed metabolites in three chitosan fragment treatments, we further conducted a pathway analysis and the results are displayed in Supplementary Figures S2A–C. It seemed that the pathways related to primary C and N metabolism in (GlcN)_7_ treatment groups had a higher pathway enrichment and higher pathway impact values than those in (GlcN)_6_ and (GlcN)_8_ treatment groups.

### Impact of Chitosan on Photosynthetic C Metabolism of Wheat Seedlings

**FIGURE 3:**
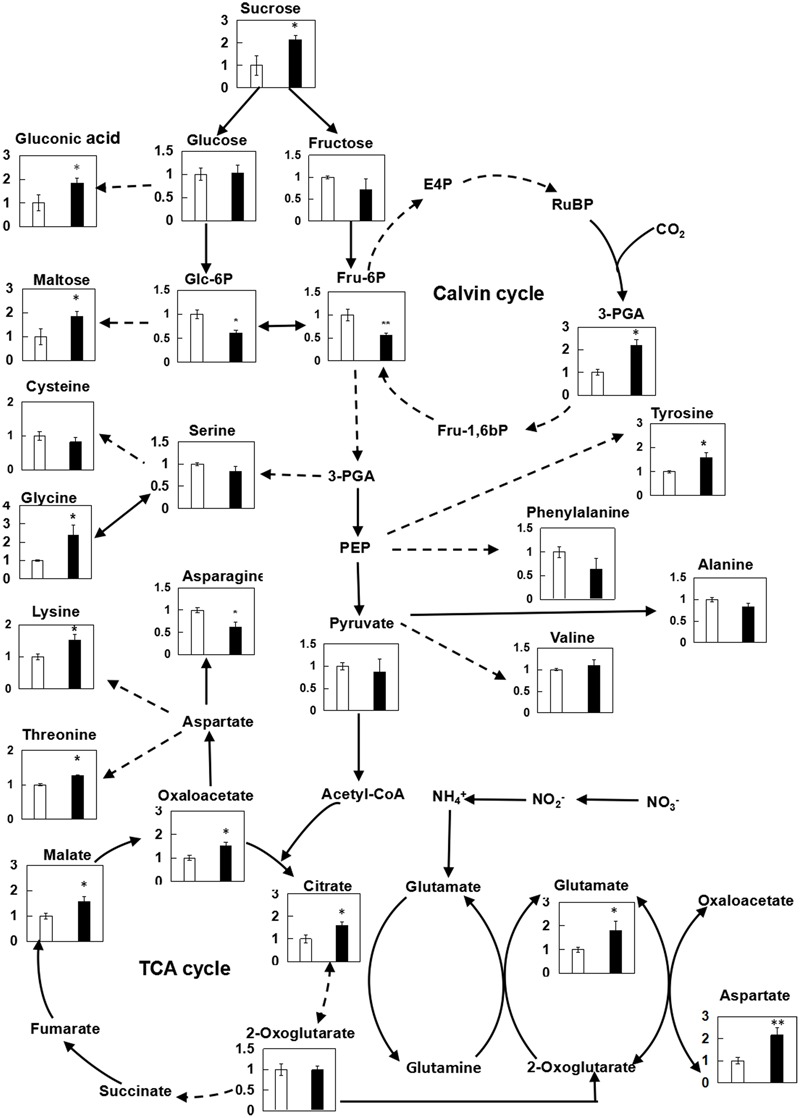
Relative contents of metabolites in leaves of wheat seedlings treated with (GlcN)_7_ determined by GC-TOF-MS. The relative metabolite levels are normalized to internal standard (ribitol) and fresh weight (FW) of samples, and then expressed as the ratio between values obtained from CK and (GlcN)_7_ -treated groups, and ultimately displayed on a primary metabolite map. Continuous arrows represent a single enzymatic step while broken arrows represent pathways involving more than one reaction. The values are mean ± SE calculated from six independent biological replicates. Significant differences from the control are shown (^∗^*P* < 0.05, ^∗∗^*P* < 0.01; *t*-test).

**FIGURE 4:**
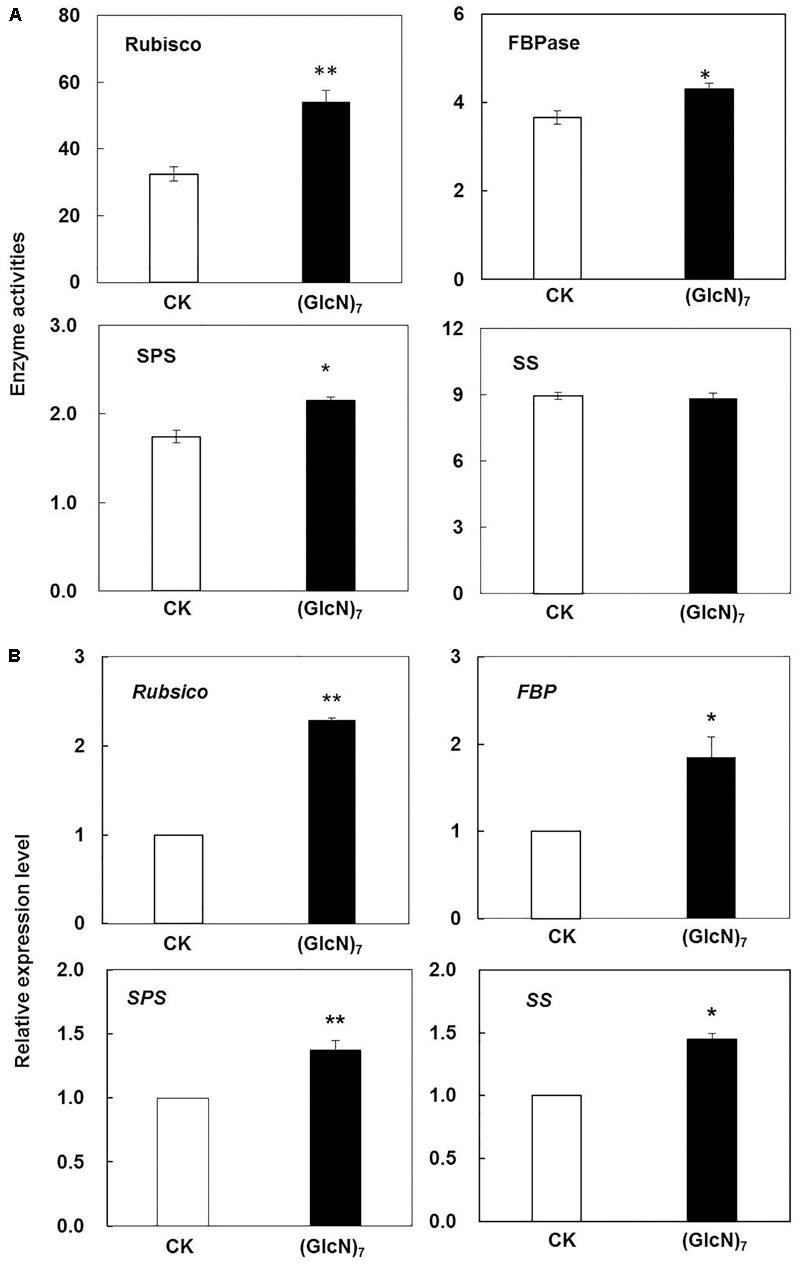
Impacts of (GlcN)_7_ on the photosynthetic C metabolism of wheat seedlings. **(A)** Key enzyme activities in photosynthetic C metabolism. Rubisco and FBPase activities are expressed as nmol s^-1^ g^-1^ fresh weight (FW), and SPS and SS activities are expressed as μmol min^-1^ g^-1^ FW. **(B)** Relative expression level of key enzymes in photosynthetic C metbolism. Relative expression levels are calculated and normalized using β*-actin* as an internal control. Each value represents means ± SD calculated from four independent biological replicates. Significant differences from the CK are shown (^∗^*P* < 0.05, ^∗∗^*P* < 0.01; *t*-test).

In present study, the effect of chitosan fragment on the fluorescence emission spectra of wheat leaves was further explored (Supplementary Figure S3). During experiment as compared with the control, the relative fluorescence intensity at 682 nm, which was rooted in the light-harvesting complex of PSII, was increased by 31.8%. Correspondingly, the effects of (GlcN)_7_ on metabolites involved in photosynthetic C metabolism were also displayed in **Figure 3**. Compared with the control, Glc-6P and Fru-6P levels were both decreased, while significant improvements of sucrose, maltose, gluconic acid, and 3-PGA contents were observed in (GlcN)_7_-treated group (*P* < 0.05), which implied chitosan may activate the photosynthetic C metabolism. Accordingly, the related enzymes activities and their transcript levels in C assimilation were further measured to verify the promoting effect of chitosan on the photosynthetic C metabolism of wheat seedlings (Stitt and Hurry, 2002). Rubisco could catalyze the carboxylation of RuBP and produce 3-PGA. The current experimental results showed that the Rubisco activity was significantly increased by 66.4% (**Figure 4A**) and this improvement was consistent with the increase of 3-PGA content (**Figure 3**), proving that chitosan really enhanced the fixation of CO_2_. On the other hand, as an end-product of photosynthesis, sucrose content was also increased by 1.1-fold in (GlcN)_7_-treated leaves (**Figure 3**), and this improvement was correlated with the enhanced activity of SPS and fructose-1,6-bisphosphatase (FBPase), which were increased by 17.5 and 29.4%, respectively. However, the sucrose synthetase (SS) activity in (GlcN)_7_ treatment group showed no significant difference. Importantly, the Rubisco, FBPase, SPS, and SS activities in response to chitosan were all in accordance with their changes at transcript levels (**Figure 4B**).

### Impact of Chitosan on Glycolysis and TCA Cycle of Wheat Seedlings

**FIGURE 5:**
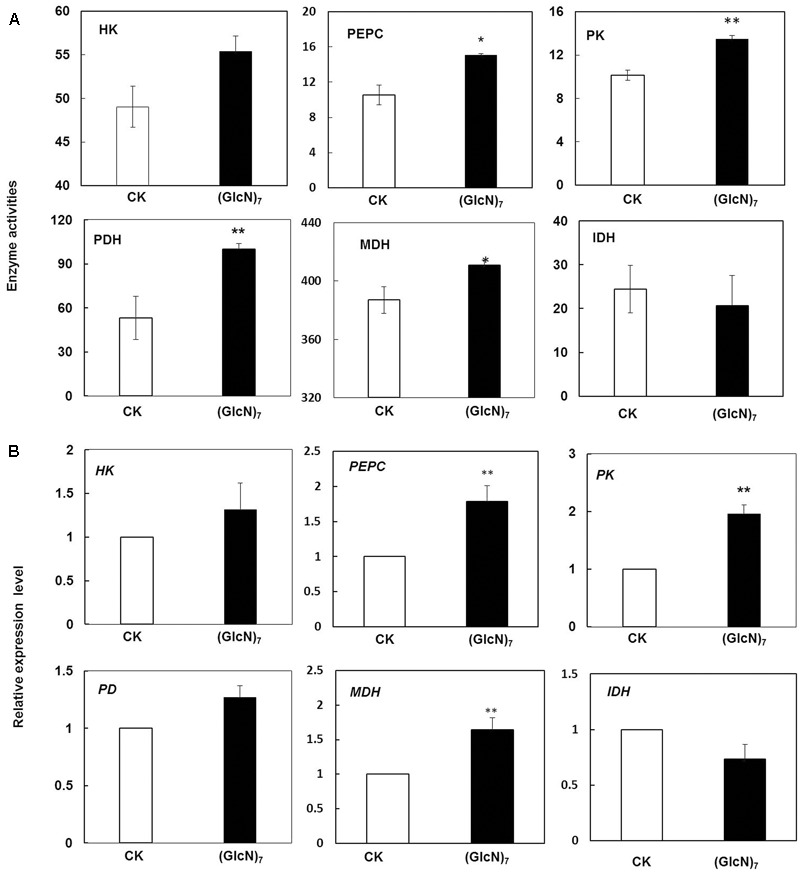
Impacts of (GlcN)_7_ on glycolysis and TCA cycle of wheat seedlings. **(A)** Key enzymes activities in glycolysis and TCA cycle. PEPC and PK activities are expressed as nmol s^-1^ g^-1^ FW and HK, PDH, MDH and IDH activities are expressed as μmol min^-1^ g^-1^ FW. **(B)** Relative expression level of key enzymes in glycolysis and TCA cycle. Relative expression levels are calculated and normalized using *β-actin* as an internal control. Each value represents means ± SD calculated from four independent biological replicates. Significant differences from the CK are shown (^∗^*P* < 0.05, ^∗∗^*P* < 0.01; *t*-test).

Based on the results obtained by metabolic profiling, effects of chitosan on relevant enzyme activities and their corresponding transcript levels in glycolysis and TCA cycle were further studied. HK had been reported to regulate the photosynthesis, growth, and senescence of rice (Cho et al., 2009). According to current study, the Glc-6P level in (GlcN)_7_ treatment group was decreased while the content of glucose had no significant change (**Figure 3**), which were paralleled with the responses of HK activity and its transcript level (**Figure 5**). Moreover, compared with the control, the contents of citrate, malate and oxaloacetate in (GlcN)_7_ treatment group were increased by 60.0, 57.0, and 51.3% (**Figure 3**), respectively, and these increases were well matched the increased phosphoenolpyruvate carboxylase (PEPC) and MDH activities (**Figure 5**). Furthermore, the intermediates in glycolysis and TCA cycle could be used as precursors for amino acid synthesis. **Figure 3** displayed that glycine content was increased along with the change of 3-PGA described above. The level of aspartate, lysine and threonine were also markedly improved, which might be associated with the enhancement of oxaloacetate level. Interestingly, asparagine content was reduced in (GlcN)_7_ treatment group. The pyruvate and 2-oxoglutarate contents did not show any obvious difference, which were paralleled to the change of alanine and valine contents (**Figure 3**). However, PK and PDH activities were obviously enhanced by 33.3 and 88.7%, respectively (**Figure 5A**), which were inconsistent with the changes of related metabolites in (GlcN)_7_ treatment group. Furthermore, at transcriptional level, the gene expression of PEPC, MDH, and PK were all significantly increased (**Figure 5B**).

### Impact of Chitosan on Primary N Metabolism of Wheat Seedlings

**FIGURE 6:**
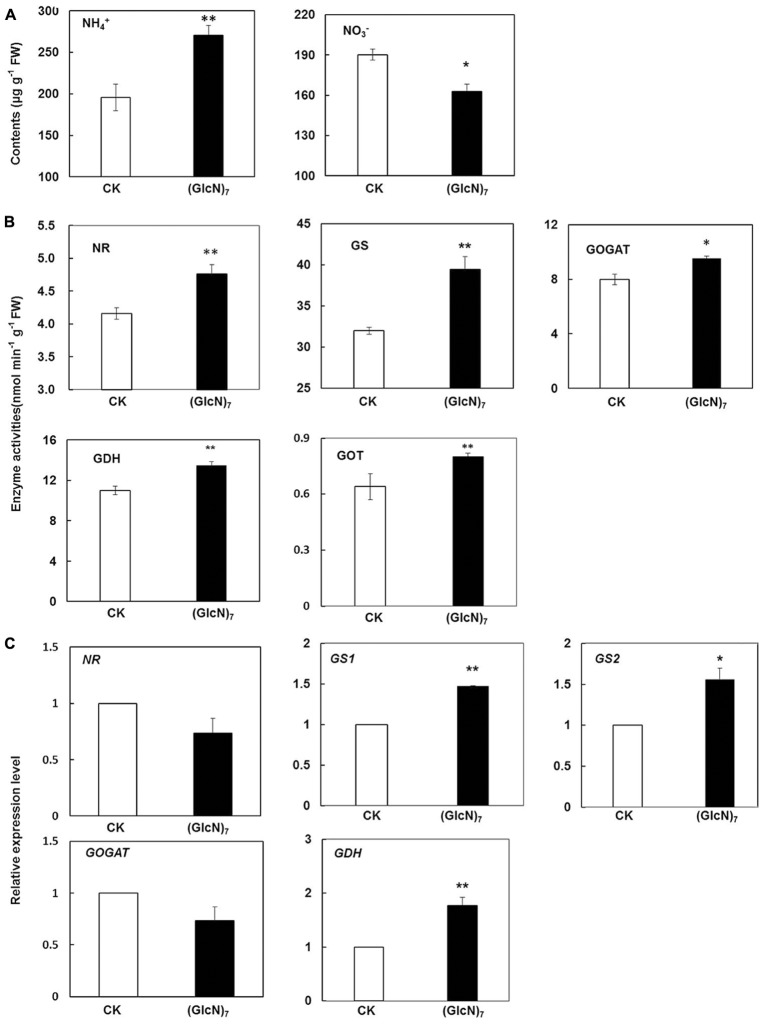
Impacts of (GlcN)_7_ on the central N metabolism of wheat seedlings. Metabolite, enzyme activities and transcript levels associated with in wheat seedlings N reduction and N assimilation were analyzed. **(A)** Contents of NH_4_^+^ and NO_3_^-^ in leaves of CK and (GlcN)_7_ treatment group. **(B)** Relative enzymes activities in N reduction and N assimilation. **(C)** Relative expression level of key enzymes in N reduction and N assimilation. Relative expression levels are calculated and normalized using *β-actin* as an internal control. Each value represents means ± SD calculated from four independent biological replicates. Significant differences from the CK are shown (^∗^*P* < 0.05, ^∗∗^*P* < 0.01; *t*-test).

In plants, nitrate reduction and subsequent N assimilation are both essential to plant growth and development. NR and nitrite reductase (NiR) could catalyze the reduction from nitrate to ammonium, which is then converted into glutamate and glutamine through GS, GOGAT, and GDH (Nunes-Nesi et al., 2010). In order to investigate the chitosan-triggered response of the central N metabolism in wheat seedlings, the contents of nitrate (NO_3_^-^) and ammonium (NH_4_^+^), key enzymes activities and their transcript levels were comprehensively measured in present study (Reguera et al., 2013). As is illustrated in **Figure 6A**, the content of NO_3_^-^ was significantly decreased while a substantial increase of NH_4_^+^ level was observed after (GlcN)_7_ treatment, which was associated with the significant improvement of NR activity (**Figure 6B**). Moreover, glutamate level was also increased by 80.0%, which was well correlated with the elevation of the GS and GOGAT activities in (GlcN)_7_ treatment group. Glutamate could be as an amino donor to synthesize aspartate via aminotransferase reactions. Correspondingly, the current study showed that (GlcN)_7_ triggered the improvement of aspartate level accompanied by increased oxaloacetate transaminase (GOT) activity (**Figures 3, 6B**). In the meanwhile, (GlcN)_7_ also stimulated the GDH activity. At transcript level, the relative expression levels of *GS1* and *GS2*, which encode the two isoforms of GS, were induced by (GlcN)_7_ with an increase of 47.3 and 56.0%, respectively. In addition, *GDH1* was also significantly increased in accordance with the enhanced GDH activity, while the transcript levels of *NR* and *GOGAT* showed no obvious difference between CK and (GlcN)_7_ treatments (**Figure 6C**). Furthermore, the level of gene expression related to nitrate transport was also examined correspondingly. *TaNRT2.1* is the only gene belonging to the NRT2 family which has so far been characterized in wheat (Taulemesse et al., 2016). NRT2.1 is a putative high-affinity nitrate transporter and its expression level is associated with NO_3_^-^ and NH_4_^+^ contents. Moreover, an NAR2-like protein actively interacts with NRT2.1 to enable functional nitrate uptake in plant (Orsel et al., 2006). The NRT2/NAR2 system was also analyzed in this case and our results showed that the transcript abundance of genes related to NRT2/NAR2 system had no significant difference between CK and (GlcN)_7_ treatment group (Supplementary Figure S4).

## Discussion

Chitosan has been recognized as a plant elicitor, which could induce a series of defense reactions by a receptor mode or dependent on their cationic property (Miya et al., 2007). It has been reported that chitosan could specifically bind to many kinds of cell membranes including tobacco, strawberry, and oilseed rape cell and its binding specificity depended on the size of chitosan fragments (Guo et al., 2009; Yin et al., 2013, 2016). In previous study (Zhang et al., 2016), it had showed that the growth-promoting effect of chitosan was closely associated with its size, and (GlcN)_6_, (GlcN)_7_, and (GlcN)_8_ were more effective in promoting plant growth than other chitosan fragments and thus were selected to be used in this study. In order to reveal the mode of action of chitosan-mediated promoting effect on plants, the metabolic responses of wheat seedlings to those three chitosan fragments, including photosynthetic carbon metabolism, glycolysis, TCA cycle and central N metabolism, were investigated via an integrative analysis of metabolites, enzyme activities and transcript levels. It is known that plant growth was closely associated with its metabolic signature. During this experiment, a comprehensive metabolite profiling study with GC-TOF-MS was performed to explore the chitosan-triggered growth-promoting effect on wheat seedlings. In current study, it was found (GlcN)_6_, (GlcN)_7_, and (GlcN)_8_ all could trigger significant responses of metabolites in photosynthetic C fixation, TCA cycle and N assimilation, implying chitosan played an important role in regulating C and N metabolism. However, the inductive effects of three chitosan fragments showed some differences. (GlcN)_7_ and (GlcN)_8_ both induced around 50 differentially changed metabolites, which were much more than (GlcN)_6_ did (Supplementary Tables S3–S5 and **Figure 1**). So, the metabolite profiles were much more affected by (GlcN)_7_ and (GlcN)_8_. Correspondingly, the differentially changed metabolites in (GlcN)_7_ treatment were mostly involved in carbon fixation in photosynthetic organisms, TCA cycle, pyruvate metabolism, and alanine, aspartate and glutamate metabolism in wheat seedlings (Supplementary Figure S2B). (GlcN)_8_ mainly induced the carbon fixation in photosynthetic organisms, starch and sucrose metabolism and galactose metabolism (Supplementary Figure S2B). However, (GlcN)_6_ mainly activated the fructose and manose metabolism (Supplementary Figure S2A). It seemed that (GlcN)_7_ was more effective in activating the metabolic response of wheat seedlings relative to primary C and N metabolism. In order to further validate the results of metabolite profiles and give a more comprehensive understanding of growth-promotion effect of chitosan, we therefore selected (GlcN)_7_ as a representative to investigate its impacts on primary C and N metabolism at metabolites, enzyme activities and transcript levels.

The present study suggested that (GlcN)_7_ could trigger a significant improvement of relative fluorescence intensity of PSII in thylakoid membrane, confirming that (GlcN)_7_ could increase energy exchange efficiency of PSII, and further increase the plant photosynthetic rate. However, the regulation of photosynthetic activity is not only dependent on accurate coordination of reactions in the thylakoid membranes and stroma but also relies on the accumulation of photosynthetic assimilates in leaves (Paul and Foyer, 2001). The Calvin cycle is the primary pathway for carbon fixation, and the first step of this cycle is catalyzed by the enzyme Rubisco and led to the formation of 3-PGA (Tamoi et al., 2005). 3-PGA is a key intermediate in Calvin cycle and had a positive correlation with plant growth rate (Meyer et al., 2007). (GlcN)_7_ could induce the activation of Rubisco activity and the increase of 3-PGA level in wheat seedlings (**Figures 3, 4**), which were associated with its growth-promoting effect. Furthermore, the key regulatory steps of sucrose biosynthesis are considered to be the coordination of FBPase and SPS. FBPase could catalyze the synthesis of Fru-6P, which then acts as a substrate for sucrose synthesis via SPS (Rueda-Lopez et al., 2015). The increase or decrease of the activities of these two particular enzymes directly affects the accumulation of sucrose. It has been reported that the overexpressing of cytosolic FBPase and SPS both resulted in the accumulation of sucrose in plant (Tamoi et al., 2005). In present study, the increase of sucrose content was observed in (GlcN)_7_-treated wheat seedlings, which could be attributed to the observed increase in FBPase and SPS activities. Furthermore, Fru-6P had a negative correlation with plant biomass (Meyer et al., 2007), so the reduced level of Fru-6P also confirmed that chitosan stimulated the accumulation of biomass. Therefore, chitosan could enhance the photosynthetic CO_2_ fixation and the accumulation of photosynthetic assimilates, which further contributes to the improvement of the photosynthesis and growth of wheat seedlings.

There was an interesting observation found during this study that the pyruvate level was not paralleled by the trend of enzyme activities which were directly involved in its metabolism (**Figures 3, 4**). In many cases, changes in enzyme levels did not result in significant metabolic differences, which were probably due to the coordination of interconnected network (Shi et al., 2015). Pyruvate was an essential metabolite linked glycolysis and TCA cycle together, which was synthesized via the catalysis of PK and then converted into acetyl-CoA via PDH irreversibly. Therefore, the unchanged level of pyruvate in chitosan treatment may be explained by its rapid metabolism mediated by the activated PK and PDH activities.

Conventionally, TCA cycle begins with the synthesis of citrate using oxaloacetate and acetyl-CoA as substrates and proceeds via a series of oxidative reactions, and ends with the regeneration of oxaloacetate. However, prior isotope labeling experiments had revealed that while the TCA cycle operated as a cycle during the night, the flux distribution in the illuminated leaf was mainly non-cyclic, including two pathways operating in opposing directions. One was from citrate to 2-oxoglutarate. The other pathway was from oxaloacetate to malate to fumarate, and the oxaloacetate was derived from the carboxylation of PEP (Tcherkez et al., 2009; Sweetlove et al., 2010). Actually, PEPC is a vital enzyme of primary C metabolism, and its fundamental function is not only to supply TCA intermediates by irreversibly catalyzing the reaction from PEP to cytosolic oxaloacetate but also to regulate the balance of C and N metabolism (Shi et al., 2015). The PEPC activity in (GlcN)_7_ treatment group was improved significantly, which may contribute to the increase of oxaloacetate content and integrative coordination of C and N metabolism. So (GlcN)_7_ stimulated the net accumulation of organic acids that could act as C-skeleton for the synthesis of amino acids in nitrogen assimilation. Furthermore, considering MDH activity was also activated by (GlcN)_7_ while the IDH showed no significant difference, chitosan may stimulate the TCA cycle mainly by affecting the steps of from oxaloacetate to malate to fumarate (**Figures 3, 5**).

NRT2.1, an important high-affinity nitrate transporter, could interact with an NAR2-type protein for a functional high-affinity transport system (HATS) based on the essential role of NAR2.1 (Saia et al., 2015). In particular, our results demonstrated that (GlcN)_7_ did not activate the genes expression of NRT2/NAR2 system related to nitrate transport (Supplementary Figure S4), which indicated that the decrease of NO_3_^-^ content was mainly resulted from the conversion from nitrate to ammonium via NR. Actually, the decrease of NO_3_^-^ content and the increase of NH_4_^+^ content were consistent with the level of NR activity, suggesting that (GlcN)_7_ could activate the nitrate reduction (**Figure 6**). Furthermore, GS/GOGAT pathway was considered as the main pathway of ammonia assimilation. In present study, the GS/GOGAT pathway was significantly activated by (GlcN)_7_, which could be ascribed to the elevated supply of NH_4_^+^, promotion of *GS1* and *GS2* transcripts levels, and improvements of GS, GOGAT, and GDH activities (**Figures 3, 6**). Actually, chitosan-induced promotion effect on nitrogen assimilation required a source of 2-oxoglutarate, which played a key role as primary C-acceptor in GS/GOGAT pathway for glutamate family amino acid synthesis (Hodges, 2002). It is interesting that the 2-oxoglutarate level was not changed with the activation of GS/GOGAT pathway in chitosan treatment, which may reflect a rapid metabolism that leads to a balance of production and consumption of 2-oxoglutarate in leaves of wheat seedlings. The 2-oxoglutarate in plant cell could be produced by some metabolic pathways and enzymatic reactions, such as the decarboxylation of isocitrate catalyzed by IDH, the transamination between glutamate and aspartate, and the deamination mediated by GDH. Although GDH was involved in the ammonium assimilation, prior studies also confirmed that the main physiological function of NADH-GDH was to provide 2-oxoglutarate for the TCA cycle and NADH-GDH played a crucial role in plant C metabolism from glutamate (Foyer et al., 2011; Fontaine et al., 2012). The activity of GDH and its transcript level were also activated by (GlcN)_7_, which may contribute to the supply of 2-oxoglutarate. Moreover, the activation of transamination via GOT provided the 2-oxoglutarate for nitrogen assimilation as well. However, the IDH activity showed no significant difference between the CK and chitosan treatment. It could be explained by the evidence that the production of 2-oxoglutarate by the NAD-dependent IDH was not limiting for N assimilation (Lemaitre et al., 2007). Another noteworthy observation is that the asparagine level in chitosan treatment was reduced. It was well known that asparagine, carrying an extra nitrogen atom in its side chains, was regarded as an important nitrogen carrier in cellular metabolism. So, the reduced levels of asparagine in chitosan treatment implied that it was utilized more frequently when plant growth was stimulated by the (GlcN)_7_.

The C and N metabolism are tightly associated with each other. Most importantly, numerous studies had provided the evidences that NR and PEPC are two of the major metabolic checkpoints coordinating primary N and C assimilation in plants (Foyer et al., 2003). NR was considered as a regulator of the influx of inorganic nitrogen into nitrogen assimilation and PEPC regulated the organic acid production. We found that the NR and PEPC in leaves of wheat seedlings were both activated by chitosan fragment, implying that chitosan, to some extent, could promote the coordination of the C and N metabolism.

In summary, the integrative analysis of metabolites, enzyme activities and transcript levels revealed that chitosan could regulate a series of primary C and N metabolic pathways in leaves of wheat seedlings. The photosynthetic C fixation was enhanced by chitosan with a higher accumulation of sucrose. Chitosan also triggered the increase of organic acids, such as oxaloacetate and malate, which could supply C skeletons for the synthesis of amino acids. Correspondingly, chitosan enhanced the N reduction and GS/GOGAT cycle. Glutamate, aspartate and some other amino acids were higher, accompanied by the activation of key enzymes of N metabolism in chitosan-treated plants. The present findings highlight the important role of chitosan in regulating the carbon and nitrogen metabolism in wheat seedlings, which will provide a new insight for understanding the mechanism of chitosan-induced growth-promoting effect and give a basic guidance for the future application of chitosan in agriculture.

## Author Contributions

KL conceived this study and designed the scientific objectives and prepared the chitosan fragments sample. XZ carried out the plant experiments and prepared the manuscript. XZ and KL performed the metabolite profiling data analysis. RX and SL supervised the project. KL and PL provided financial support and revised the paper.

## Conflict of Interest Statement

The authors declare that the research was conducted in the absence of any commercial or financial relationships that could be construed as a potential conflict of interest.

## Abbreviations

3-PGA: 3-phosphoglycerate
E4P: erythrose-4-phosphate
FBPase: Fructose 1, 6-2 phosphatase
Fru-1,6bP: Fru-1,6-bisphosphate
GDH: glutamate dehydrogenase
GOGAT: glutamate synthase
GOT: glutamate oxaloacetate transaminase
GS: glutamine synthetase
HK: hexokinase
IDH: isocitrate dehydrogenase
MDH: malate dehydrogenase
NR: nitrate reductase
OPLS-DA: orthogonal to partial least squares discriminant analysis
PCA: principal component analysis
PDH: pyruvate dehydrogenase
PEP: phosphoenolpyruvate
PEPC: phosphoenolpyruvate carboxylase
PK: pyruvate kinase
Rubisco: Rribulose-1,5-bisphosphate carboxylase/oxygenase
RuBP: ribulose-1,5-bisphosphate
SPS: sucrose phosphate synthase
SS: sucrose synthase
TCA: tricarboxylic acid
