# Supplementary material for: Metabolite Profiling of Wheat Seedlings Induced by Chitosan: Revelation of the Enhanced Carbon and Nitrogen Metabolism
Source: Front Plant Sci. 2017 Nov 28;8:2017. doi: 10.3389/fpls.2017.02017 (PMC5712320; doi:10.3389/fpls.2017.02017)
Supplement: Supplementary file 1 [file Table_1.PDF]

Supplementary Table S1 DNA sequences of PCR primers were used for qPCR determination in wheat seedlings.

Gene	GenBank Accession No.	Primer pairs
TaRBC	306885	F[a]:CAGCAACGGTGGAAGGAT<br>R[b]:GGTGGCAAGTAGGACAGG
TaFBP	277182	F:CTCCATCTTCGGCATCTACA<br>R:TCTGCGTCACTTCGTCAA
TaSS	291727	F:CCGACAAGGAGAAGTATG<br>R:CGAGTTCACTAACATTCAC
TaSPS	277083	F:ATCGTCACGCTCGCTCAA<br>R:AGTCATCTTCCTGCCAAAATTACA
TaPK	277705	F:CAAGTGGTGAACAAGAGTGAAG<br>R:AGTCCAGCGAAGTTGATAGG
TaHK	AY974231.1	F:AAACGTGCTGTCCAACAATATG<br>R:TCGTCTCCTTCAGTCTCAACAA
TaPD	GU211251.1	F:TGACCCCTTGTGTTTCTGTATC<br>R:GCATACATCCTGGGACAAAACT
TaPEPC	AJ007705	F:CAGGGTGAAGTTATCGAGCAG<br>R:ACTTGGCTTTCTCTTGGATGG
TaMDH	AK333412	F:CGATTGCTGCTGAGATTC<br>R:CAGCGTTCTGTATCCTCT
TaICDH	GR304944.1	F:TTCCGTGTTCACCAGAAAGG<br>R:GCTTCCTCAAGTTTCTGTGC
TaNR	AK333426	F:GAAAGGATACGCATACTCCGG<br>R:CGTACTTGTTCGGCTTCTCC
TaGS1	DQ124211	F:CAACCCTGATGTTGCCAAG<br>R:GTAGGCGGCGATGTGCT
TaGS2	DQ124212	F:ATTTCGAAGCCAGTGGAG<br>R:GCACTTGTGCAGTGACCTTG
TaGOGAT	KC960545	F:AGGAGATTGAAGGATCACAAGAG<br>R:GCTTTGAAGTTGGAACGGTTG
TaGDH1	HQ658905	F:ATTTCGTCGAACAGGATGAACGC<br>R:CGGCCGGCGGTCGATCAGGCCTC
TaNRT2.1	AF288688	F:ACAAGCTGCTTGTGGTGCTGTA<br>R:GTGATGAACAGTAAAATTCTTAGGTG
TaNAR2.1	AY763794	F:TCCTCTCTTGCCTTCTCCGATCGACAA<br>R:TGCACCCGAACATTTATCCGTACAGTT
TaNAR2.2	AY763795	F:TTCTCTGACCGAGCAGCTGCGAGCT<br>R:GGAAACACCAACACTCTCTTACATT
β-actin	AB181991	F:CTCTGACAATTTCCCGCTCA<br>R:ACACGCTTCCTCATGCTATCC

[a]: Forward primer

[b]: Reverse primer
