# Supplementary material for: Metabolite Profiling of Wheat Seedlings Induced by Chitosan: Revelation of the Enhanced Carbon and Nitrogen Metabolism
Source: Front Plant Sci. 2017 Nov 28;8:2017. doi: 10.3389/fpls.2017.02017 (PMC5712320; doi:10.3389/fpls.2017.02017)
Supplement: Supplementary file 2 [file Table_2.PDF]

Supplementary Table S2. Effects of chitosan on growth parameters of wheat seedlings. Values are the mean ± SD of three replicates. Different letters indicate significant differences at $P < 0.05$.

Treatment	Fresh weight (g)	Dry weight (g)	Height (cm)
CK	0.67 ±0.019b	0.075 ±0.005b	23.39 ±0.472a
$(GlcN)_6$	0.77 ±0.023b	0.08 ±0.004b	23.41 ±0.186a
$(GlcN)_7$	0.86 ±0.013a	0.10 ±0.007a	23.11 ±0.267a
$(GlcN)_8$	0.80 ±0.014a	0.11 ±0.010a	22.97 ±0.393a
