# Supplementary material for: Metabolite Profiling of Wheat Seedlings Induced by Chitosan: Revelation of the Enhanced Carbon and Nitrogen Metabolism
Source: Front Plant Sci. 2017 Nov 28;8:2017. doi: 10.3389/fpls.2017.02017 (PMC5712320; doi:10.3389/fpls.2017.02017)
Supplement: Supplementary file 3 [file Table_3.PDF]

Supplementary Table S3. List of significantly changed metabolites in the $(GlcN)_6$ treatment group.

| Metabolites name | Similarity | R.T.[a] | Mass | VIP[b] | P-value[c] | FC[d] |
|---|---|---|---|---|---|---|
| 2-Hydroxypyridine | 847 | 8.86 | 152 | 1.826 | 0.020 | 1.239 |
| Aspartic acid | 824 | 15.48 | 232 | 2.049 | 0.001 | 1.745 |
| Valine | 815 | 11.41 | 144 | 1.645 | 0.046 | 0.693 |
| Xylose | 791 | 17.10 | 103 | 2.191 | 0.005 | 0.592 |
| Threonic acid | 781 | 15.89 | 292 | 1.745 | 0.025 | 1.315 |
| Hydroxylamine | 740 | 9.98 | 146 | 2.193 | 0.002 | 0.737 |
| Itaconic acid | 732 | 13.21 | 147 | 1.784 | 0.042 | 2.165 |
| 2-Methylfumarate | 681 | 13.99 | 184 | 1.928 | 0.019 | 1.850 |
| D-Altrose | 638 | 19.78 | 273 | 1.975 | 0.037 | 2.628 |
| Dioctyl phthalate | 614 | 25.49 | 149 | 2.414 | 0.000 | 0.523 |
| 1,5-Anhydroglucitol | 598 | 19.21 | 259 | 1.873 | 0.014 | 0.072 |
| 5-Methoxytryptamine | 572 | 24.79 | 174 | 1.053 | 0.043 | 0.509 |
| Erythrose | 532 | 14.34 | 201 | 1.342 | 0.001 | 3.297 |
| Leucine | 525 | 12.21 | 158 | 2.251 | 0.037 | 3.931 |
| alpha-D-glucosamine 1-phosphate | 504 | 18.86 | 204 | 1.865 | 0.025 | 0.729 |
| 3-Phosphoglycerate | 467 | 18.71 | 299 | 1.721 | 0.046 | 2.240 |
| 4-Hydroxyquinazoline | 429 | 15.81 | 217 | 2.580 | 0.000 | 0.669 |
| 3-Hexenedioic acid | 373 | 15.39 | 221 | 1.954 | 0.012 | 0.823 |
| Glutaric acid | 372 | 14.13 | 101 | 2.025 | 0.016 | 1.910 |
| D-erythronolactone | 367 | 16.52 | 217 | 2.177 | 0.003 | 0.770 |
| 2,6-Diaminopimelic acid | 334 | 19.32 | 174 | 1.939 | 0.014 | 0.693 |
| Asparagine | 323 | 16.67 | 146 | 1.242 | 0.044 | 0.574 |
| Phthalic acid | 311 | 17.63 | 128 | 1.138 | 0.030 | 3.236 |
| Loganin | 305 | 28.05 | 199 | 1.867 | 0.028 | 0.000 |
| Xanthosine | 304 | 26.24 | 160 | 2.333 | 0.000 | 0.315 |
| Pyrrole-2-Carboxylic Acid | 299 | 13.45 | 241 | 1.375 | 0.044 | 6.508 |
| L-Dithiothreitol | 296 | 16.52 | 332 | 1.758 | 0.004 | 11.187 |
| 3,5-Dihydroxyphenylglycine | 189 | 20.51 | 383 | 1.755 | 0.016 | 3.593 |
| Gallic acid | 105 | 20.30 | 71 | 1.644 | 0.025 | 1.444 |

[a]R.T. represents retention time.

[b]VIP represents variable importance projection, metabolite (VIP > 1) was listed in table.

[c]P-values were calculated according to Student's T-test and

[d]FC represents the fold change of the peak intensity for the $(GlcN)_6$ group against the CK (n = 6).
