# Supplementary material for: Metabolite Profiling of Wheat Seedlings Induced by Chitosan: Revelation of the Enhanced Carbon and Nitrogen Metabolism
Source: Front Plant Sci. 2017 Nov 28;8:2017. doi: 10.3389/fpls.2017.02017 (PMC5712320; doi:10.3389/fpls.2017.02017)
Supplement: Supplementary file 4 [file Table_4.PDF]

Supplementary Table S4. List of significantly changed metabolites in the $(GlcN)_7$ treatment group.

Metabolites name	Similarity	R.T.[a]	Mass	VIP[b]	P-value[c]	FC[d]
Glycine	863	12.72	174	1.676	0.049	1.811
D-Glyceric acid	837	12.97	189	1.389	0.016	1.501
Maltose	825	26.80	160	1.882	0.035	1.831
Citric acid	824	18.84	273	1.290	0.038	1.600
Threonine	814	13.74	219	1.420	0.037	1.263
Aspartic acid	824	15.48	232	1.692	0.013	1.695
Lysine	792	19.96	156	1.830	0.023	1.509
L-Malic acid	783	15.05	73	1.333	0.043	1.400
Threonic acid	781	15.89	292	1.957	0.004	1.322
Glucose-6-phosphate	764	23.53	387	2.034	0.004	0.612
Sucrose	764	26.08	455	1.901	0.039	2.004
Glutamic acid	756	16.66	246	1.610	0.040	1.799
Glycolic acid	751	9.35	147	1.577	0.038	1.307
Hydroxylamine	740	9.98	146	2.195	0.000	0.458
Xylitol	662	17.52	103	1.423	0.046	0.837
Sedoheptulose	648	20.17	204	1.404	0.046	0.642
Saccharic acid	618	20.96	333	1.783	0.000	3.220
Dioctyl phthalate	614	25.49	149	2.348	0.000	0.505
1,5-Anhydroglucitol	598	19.22	259	1.771	0.008	0.098
Fructose-6-phosphate	578	23.41	315	1.989	0.008	0.462
Trehalose-6-phosphate	561	29.73	204	1.297	0.005	0.242
Guanidinosuccinic acid	547	17.44	328	1.322	0.033	0.610
Diglycerol	535	18.25	292	1.897	0.015	1.528
Erythrose	532	14.34	201	1.287	0.000	3.757
alpha-D-glucosamine 1-phosphate	504	18.86	204	1.820	0.017	0.738
3-Phosphoglycerate	467	18.71	299	1.921	0.011	2.210
Allose	466	16.95	160	2.024	0.007	2.067
L-Threose	451	14.77	173	1.669	0.024	1.448
4-Hydroxyquinazoline	429	15.81	217	2.270	0.000	0.648
3-Hydroxybutyric acid	427	10.67	281	1.736	0.021	1.246
3-Hydroxypropionic acid	417	10.33	133	2.028	0.002	1.481
N-Acetyl-L-aspartic acid	414	17.27	173	2.119	0.001	1.389
Oxalacetic acid	401	14.89	173	1.749	0.018	1.513
2,4-Diaminobutyric acid	393	16.73	160	1.994	0.005	1.866
Ornithine	393	18.92	174	2.010	0.011	1.542
3-Hexenedioic acid	373	15.39	221	1.677	0.026	0.822
Glutaric acid	372	14.13	101	2.102	0.003	2.350
D-erythronolactone	367	16.52	217	1.631	0.048	0.846
Palmitoleic acid	344	21.11	73	1.646	0.027	1.379

Luteolin	334	30.46	345	1.768	0.015	1.414
3-Isochromanone	328	15.70	292	1.745	0.019	1.506
Asparagine	323	16.68	146	1.680	0.030	0.610
beta-Hydroxymyristic acid	318	21.05	199	1.316	0.069	0.703
Xanthosine	304	26.24	160	2.098	0.001	0.432
alpha-Ecdysone	301	32.24	249	1.857	0.014	1.545
Pyrrole-2-Carboxylic Acid	299	13.45	241	2.210	0.000	9.095
L-Dithiothreitol	296	16.52	332	1.792	0.005	9.953
2-Hydroxy-3-isopropylbutanedioic acid	273	16.31	229	1.431	0.012	5.138
2-Ketoadipate	272	11.65	159	1.730	0.018	0.187
Lactamide	257	10.34	267	1.170	0.047	0.562
Butyraldehyde	243	12.53	341	1.558	0.018	0.395
Leucrose	243	27.44	211	1.485	0.043	1.215
alpha-Aminoadipic acid	199	17.67	272	1.444	0.024	6.997
Gallic acid	105	20.29	71	1.172	0.018	0.446
4-Acetamidobutyric acid	79	15.46	305	1.709	0.023	0.208

[a]R.T. represents retention time.

[b]VIP represents variable importance projection, metabolite (VIP > 1) was listed in table.

[c]P-values were calculated according to Student’s T-test and

[d]FC represents the fold change of the peak intensity for the $(GlcN)_7$ group against the CK (n = 6).
