# Supplementary material for: Metabolite Profiling of Wheat Seedlings Induced by Chitosan: Revelation of the Enhanced Carbon and Nitrogen Metabolism
Source: Front Plant Sci. 2017 Nov 28;8:2017. doi: 10.3389/fpls.2017.02017 (PMC5712320; doi:10.3389/fpls.2017.02017)
Supplement: Supplementary file 5 [file Table_5.PDF]

Supplementary Table S5. List of significantly changed metabolites in the $(GlcN)_8$ treatment group.

Metabolites name	Similarity	R.T.[a]	Mass	VIP[b]	P-value[c]	FC[d]
2-Hydroxypyridine	847	8.86	152	1.791	0.008	1.303
Myo-inositol	839	21.59	191	1.694	0.023	0.815
Maltose	825	26.80	160	1.557	0.014	2.003
Ethanolamine	827	12.16	174	1.508	0.046	0.915
3,4-Dihydroxycinnamic acid	827	22.03	219	1.475	0.043	1.345
Aspartic acid	824	15.48	232	1.654	0.008	1.610
Fructose	820	19.41	103	1.657	0.022	0.918
Xylose	791	17.10	103	1.599	0.027	0.709
Aconitic Acid	784	18.12	229	1.862	0.029	2.420
L-Malic acid	783	15.05	73	1.510	0.036	1.504
Shikimic acid	772	18.70	204	1.724	0.045	2.587
Sucrose	764	26.08	455	1.748	0.005	2.504
Hydroxylamine	740	9.98	146	1.550	0.017	0.540
Glucose-1-phosphate	706	18.30	217	1.992	0.002	0.784
Tagatose	680	19.21	173	1.779	0.045	1.560
Linolenic acid	647	22.78	79	1.403	0.027	0.431
D-Altrose	638	19.78	273	1.806	0.046	2.672
Gluconic acid	626	20.82	204	1.399	0.035	0.681
Saccharic acid	618	20.95	333	1.731	0.001	4.404
Dioctyl phthalate	614	25.49	149	1.525	0.025	0.701
5-Methoxytryptamine	572	24.79	174	1.311	0.042	0.647
5'-Methylthioadenosine	563	27.28	236	1.538	0.042	0.684
Phenyl beta-D-glucopyranoside	550	23.45	204	1.798	0.010	0.560
Fructose 2,6-biphosphate	547	22.64	227	1.739	0.008	0.643
D-Talose	545	19.87	244	1.537	0.023	2.453
Erythrose	532	14.34	201	1.112	0.011	2.371
Fucose	472	18.03	174	1.785	0.005	0.628
3-Phosphoglycerate	467	18.71	299	2.221	0.036	2.271
4-Hydroxycinnamic acid	449	20.27	293	2.492	0.000	0.000
Methylmalonic acid	443	11.27	147	2.111	0.033	0.275
Maleic acid	431	12.61	306	1.590	0.035	0.832
4-Hydroxyquinazoline	429	15.81	217	1.957	0.004	0.704
1-Kestose	426	31.14	242	1.424	0.027	0.743
3-Hydroxypropionic acid	417	10.33	133	2.120	0.000	1.596
Oxalacetic acid	401	14.89	173	1.893	0.039	1.150
3-Hexenedioic acid	373	15.39	221	1.780	0.008	0.764
Glutaric acid	372	14.13	101	1.402	0.050	1.800
D-Erythronolactone	367	16.52	217	1.749	0.019	0.807
N-Ethylglycine	361	11.14	147	1.590	0.046	0.776

Ribose-5-phosphate	342	21.70	389	1.511	0.044	2.184
Maleamate	335	15.85	219	1.674	0.019	1.430
Xanthosine	304	26.24	160	2.311	0.001	0.293
Pyrrole-2-Carboxylic Acid	299	13.45	241	2.103	0.000	9.198
L-Dithiothreitol	296	16.52	332	2.138	0.000	10.985
Prostaglandin A2 3	272	26.39	93	1.244	0.004	0.343
N-Methylanthranilic acid	263	15.94	209	1.464	0.040	0.753
o-Hydroxyhippuric acid	238	21.48	181	1.784	0.005	2.153
D-Galacturonic acid	180	20.24	175	1.504	0.003	0.306

[a]R.T. represents retention time.

[b]VIP represents variable importance projection, metabolite (VIP > 1) was listed in table.

[c]P-values were calculated according to Student's T-test and

[d]FC represents the fold change of the peak intensity for the $(GlcN)_8$ group against the CK (n = 6).
