# Supplementary material for: Metabolite Profiling of Wheat Seedlings Induced by Chitosan: Revelation of the Enhanced Carbon and Nitrogen Metabolism
Source: Front Plant Sci. 2017 Nov 28;8:2017. doi: 10.3389/fpls.2017.02017 (PMC5712320; doi:10.3389/fpls.2017.02017)
Supplement: Supplementary file 6 [file Image_1.PDF]

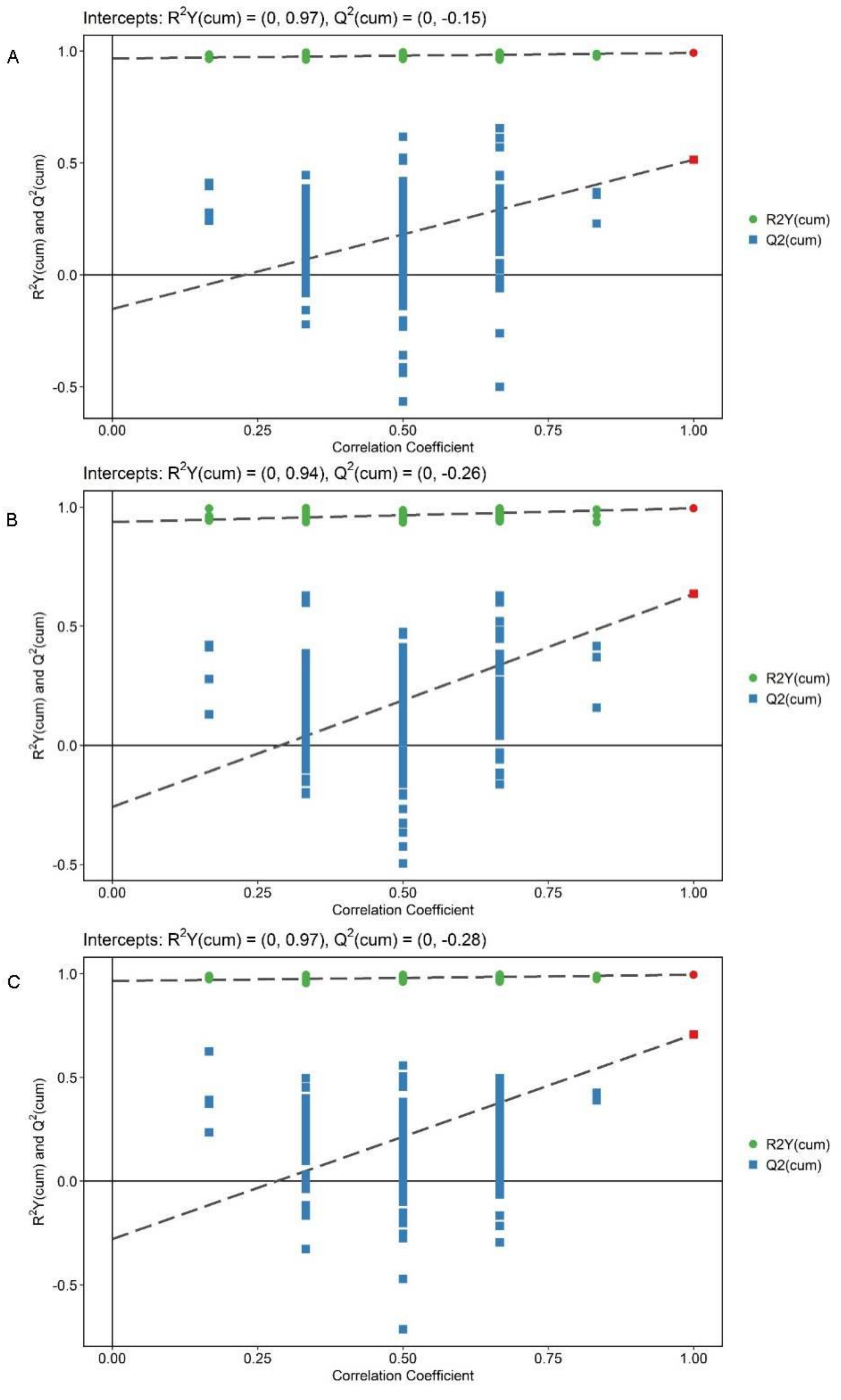

Supplementary Fig. S1. Permutation test of OPLS-DA model for (A) CK-$(GlcN)_6$, (B) CK-$(GlcN)_7$ and (C) CK-$(GlcN)_8$.
