# Supplementary material for: Metabolite Profiling of Wheat Seedlings Induced by Chitosan: Revelation of the Enhanced Carbon and Nitrogen Metabolism
Source: Front Plant Sci. 2017 Nov 28;8:2017. doi: 10.3389/fpls.2017.02017 (PMC5712320; doi:10.3389/fpls.2017.02017)
Supplement: Supplementary file 7 [file Image_2.PDF]

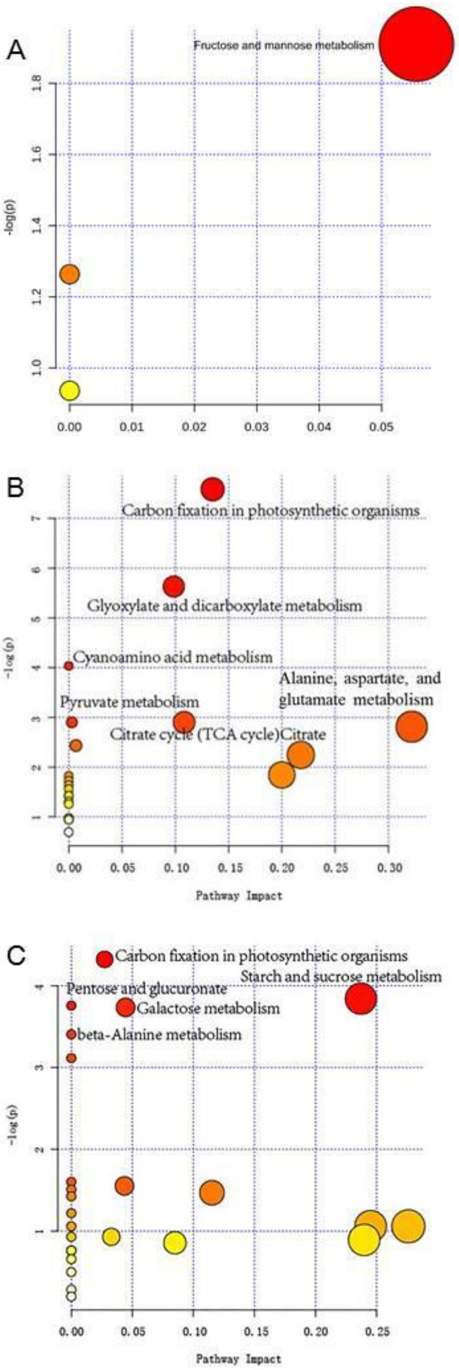

Supplementary Fig. S2. Pathway analysis plot of differentially changed metabolites for (A) CK-$(GlcN)_6$, (B) CK-$(GlcN)_7$ and (C) CK-$(GlcN)_8$. The x-axis represents the pathway impact, and y-axis represents the pathway enrichment. Larger sizes and darker colors represent higher pathway enrichment and higher pathway impact values, respectively.
