# Supplementary material for: Metabolite Profiling of Wheat Seedlings Induced by Chitosan: Revelation of the Enhanced Carbon and Nitrogen Metabolism
Source: Front Plant Sci. 2017 Nov 28;8:2017. doi: 10.3389/fpls.2017.02017 (PMC5712320; doi:10.3389/fpls.2017.02017)
Supplement: Supplementary file 8 [file Image_3.PDF]

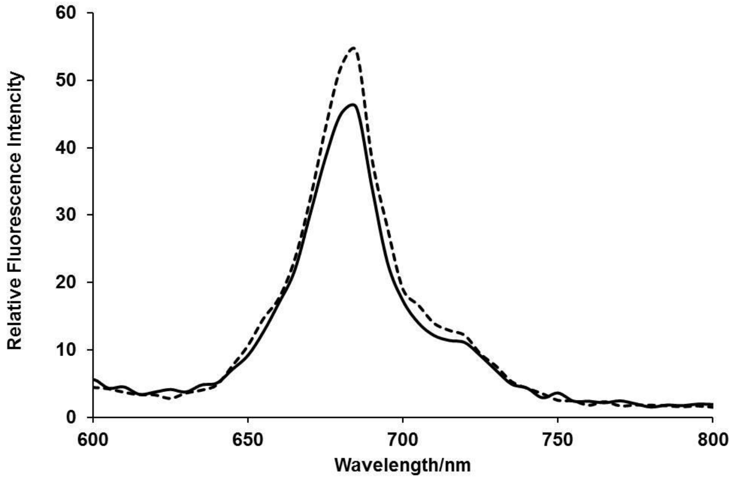

Supplementary Fig. S3 The fluorescence emission spectra in leaves of CK and $(GlcN)_7$ treatment group. The full line represents the CK, and the dotted line represents the $(GlcN)_7$ treatment group.
