# Supplementary material for: Metabolite Profiling of Wheat Seedlings Induced by Chitosan: Revelation of the Enhanced Carbon and Nitrogen Metabolism
Source: Front Plant Sci. 2017 Nov 28;8:2017. doi: 10.3389/fpls.2017.02017 (PMC5712320; doi:10.3389/fpls.2017.02017)
Supplement: Supplementary file 9 [file Image_4.PDF]

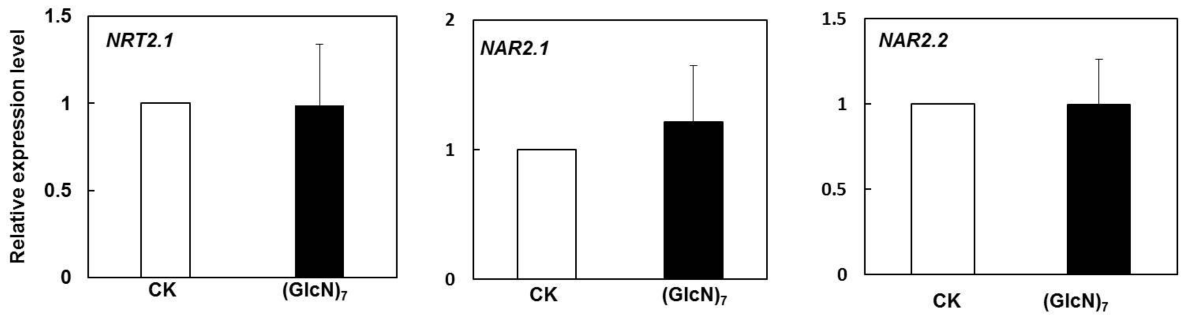

Supplementary Fig. S4 Impacts of $(GlcN)_7$ on the NRT2/NAR2 system of wheat seedlings. Relative expression levels are calculated and normalized using *β-actin* as an internal control. Each value represents means ± SD calculated from 4 independent biological replicates. Significant differences from the CK are shown (* $P < 0.05$, ** $P < 0.01$; *t* test).
